# A Review on the Synthesis and Chemical Transformation of Quinazoline 3-Oxides

**DOI:** 10.3390/molecules27227985

**Published:** 2022-11-17

**Authors:** Malose J. Mphahlele

**Affiliations:** Department of Chemistry, College of Science, Engineering and Technology, University of South Africa, Private Bag X06, Florida 1710, South Africa; mphahmj@unisa.ac.za

**Keywords:** pyrimidine, quinazoline oxides, synthesis, transformation

## Abstract

The synthesis of quinazoline 3-oxides and their derivatives has attracted considerable attention due to their reactivity as intermediates in the synthesis of quinazoline analogues and their ring-expanded derivatives. Despite this, there is no comprehensive review dedicated to the synthesis and chemical transformation of these biologically relevant azaaromatic oxides. This review aims to provide an up-to-date record of the synthesis of quinazoline 3-oxides and their chemical transformation. It is hoped that this information will help medicinal chemistry researchers to design and synthesize new derivatives or analogues to treat various diseases.

## 1. Introduction

**Figure 1:**
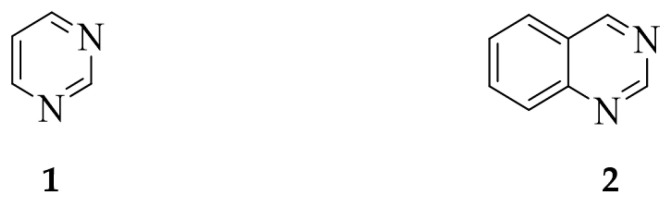
Structures of pyrimidine **1** and quinazoline **2**.

Pyrimidine **1**, shown in Figure 1, is a six-membered heterocyclic aromatic organic compound containing two nitrogen atoms at positions 1 and 3. This scaffold forms nuclei of several pharmacologically relevant compounds with a wide spectrum of biological activities, including anti-tubercular, anti-bacterial, anti-fungal, anti-viral, and anti-inflammatory properties [1]. The pyrimidine ring readily undergoes *N*-oxidation using hydrogen peroxide, *m*-chloroperbenzoic acid (MCPBA), monopermaleic acid, monoperphtalic acid, or *p*-methylperbenzoic acid to afford pyrimidine *N*-oxides [2]. However, this nucleus is susceptible to hydrolysis, ring opening, and decomposition during oxidation resulting in reduced yields of the *N*-oxide [3]. Benzo-fused pyrimidine derivatives such as quinazolines (1,3-diazanaphthalenes) **2** are also associated with a wide range of biological and pharmacological activities, including anti-cancer, anti-tuberculosis, anti-hypertensive, anti-bacterial, anti-inflammatory, and anti-malarial properties [4]. Considerable effort has been devoted to the synthesis, transformation, and biological properties of these benzo-fused pyrimidine derivatives [4,5,6,7]. Both nitrogen atoms of the pyrimidine nucleus of quinazolines can be oxidised to afford either the 1-oxide or 3-oxide derivatives. Among this class of nitrogen-based heterocycles, quinazoline 3-oxides represent valuable intermediates in the synthesis of benzodiazepine analogues [8] and other polycyclic compounds of biological importance [9]. Many valuable benzodiazepine-based drugs for the treatment of seizures and anxiety, such as chlordiazepoxide and diazepam, were first prepared from the corresponding quinazoline 3-oxides [8]. Despite this, the synthesis, transformation, and applications of the quinazoline-3-oxides have received less attention when compared to the other classes of *N*-oxides, such as 5-membered heteroaromatic *N*-oxides, pyridine *N*-oxides, and diazine *N*-oxides [8]. Interestingly, quinazoline 3-oxides do not feature in any of the reviews dedicated to the synthesis, biological activity, and chemical transformation of quinazolinones and/or quinazoline derivatives. In view of the considerable interest in quinazoline 3-oxides as bronchodilators, cardiotonics, and fungicides [10], it was decided to provide an up-to-date record of their synthesis and chemical transformation.

## 2. Methods for the Synthesis of Quinazoline Oxides

### 2.1. Direct Oxidation of Quinazolines

**Scheme 1:**
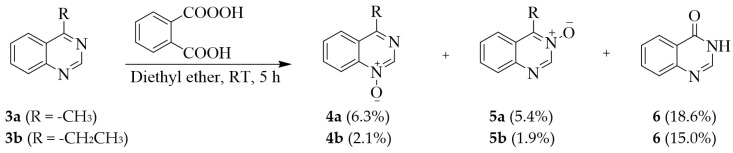
Oxidation of quinazoline scaffold.

Although there are several reagents that can be used for the direct oxidation of quinazoline nuclei to *N*-oxides, this approach is complicated by the lack of selectivity. Moreover, the pyrimidine nucleus is susceptible to hydrolysis, ring opening, and decomposition resulting in reduced yields of the *N*-oxides. Treatment of 4-alkylsubstituted quinazoline **3a** (R = -CH_3_) and **3b** (R = -CH_2_CH_3_) with monoperphthalic acid (1.2–1.3 equiv.) in ether at room temperature (RT) for 5 h, for example, afforded mixtures of the corresponding *N*-1 (**4a**, **b**) and *N*-3 oxides (**5a**, **b**) as well as the quinazolinone derivative **6** (Scheme 1) [11]. The preference for *N*-1 oxidation over the *N*-3 centre resulted in significantly reduced yields of the biologically relevant quinazoline 3-oxides. Moreover, this reaction produced quinazolin-4(3*H*)-one as the main product and quinazoline *N*-oxides as by-products. Recourse to the literature revealed a method that made use of a recombinant soluble di-iron monooxygenase (SDIMO) PmlABCDEF overexpressed in *Escherichia coli* which was used as a whole-cell biocatalyst to oxidize pyridines, pyrazines, pyrimidines, and their benzo-fused derivatives into the corresponding *N*-oxides [12]. Quinazoline **2** was among the benzo-fused heterocycles with two nitrogen atoms, which was transformed into quinazoline 3-oxide in 67% yield without any side oxidation products.

The drawback associated with the direct *N*-oxidation of quinazoline scaffold using strong oxidizing agents led to the development of alternative methods for the synthesis of quinazoline 3-oxides, and these strategies are described in detail below.

### 2.2. Synthesis of Quinazoline 3-Oxides

The most common strategy for the synthesis of quinazoline 3-oxides is based on intramolecular cyclocondensation of the intermediate *N*-acyl-2-aminoaryl ketone oximes using various reagents.

#### 2.2.1. Intramolecular Cyclocondensation of the *N*-Acyl-2-aminoaryl Ketone Oximes

**Scheme 2:**
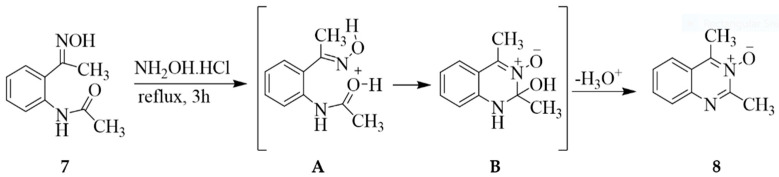
Preparation of quinazoline 3-oxides from an *N*-acyl 2-aminoaryl ketone **7**.

The 2-aminoaryl ketone oximes were previously cyclized with triethyl orthoformate to afford quinazoline 3-oxides albeit in low yields [13]. Improved yields of the 2,4-dicarbo substituted quinazoline 3-oxides were achieved via initial acylation of 2-aminoacetophenone followed by intramolecular cyclocondensation of the intermediate *N*-acyl-2-aminoaryl ketone oximes with hydroxylamine hydrochloride [14,15,16]. The *N*-oxide of 2,4-dimethylquinazoline **8**, for example, was obtained in 75% yield by treatment of (*E*)-*N*-(2-(1-(hydroxyimino)ethyl)phenyl)acetamide **7** with hydroxylamine hydrochloride under reflux for 3 h (Scheme 2) [15]. Hydroxylamine hydrochloride serves as a proton source to protonate an oxygen atom of the amide moiety, followed by cyclocondensation of the incipient intermediate **A** to afford **B**. The latter then undergoes dehydrogenation to afford the fully aromatic derivative **8**. Hitherto, the analogous 2-alkyl/cycloalkyl substituted 4-methylquinazoline 3-oxides were evaluated for biological activity as pulmonary-selective inhibitors of ovalbumin-induced, leukotriene-mediated bronchoconstriction [17]. The most active and selective compounds contained a methyl group at the 4-position, a medium-sized branched alkyl group at the 2-position, and a small electron donating group on the phenyl ring.

**Scheme 3:**
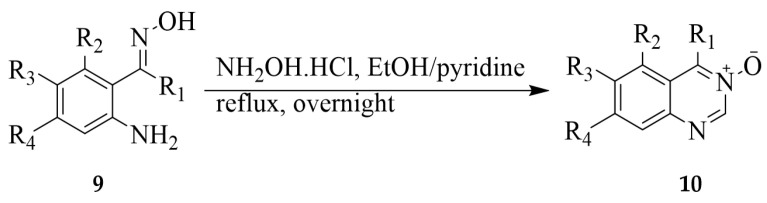
Synthesis of polysubstituted quinazoline 3-oxides.

**Scheme 4:**
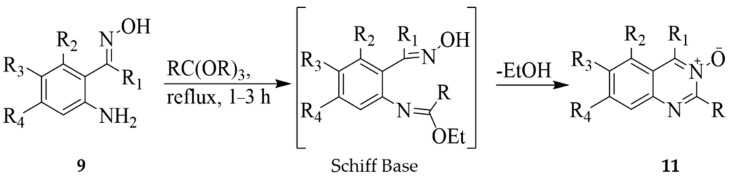
Cyclocondensation of **9** with triethyl orthopropionate or triethyl orthoacetate.

Series of quinazoline 3-oxides **10** substituted with various groups on the fused benzo ring were prepared in 12–95% yield by subjecting the 2-aminoacetophenone oxime derivatives **9** to hydroxylamine hydrochloride and pyridine-ethanol mixture under reflux (Scheme 3) [18]. The 2-carbo substituted derivatives of **11**, on the other hand, were prepared by treatment of substrates **9** with triethyl orthopropionate or triethyl orthoacetate under reflux for 1–3 h (Scheme 4). The mechanism of this reaction involves the formation of an ethoxymethyleneamino derivative or Schiff base followed by cyclocondensation to afford quinazoline 3-oxide. Analogues of compound **11** were used as cardiotonic and bronchodilating agents [18].

**Scheme 5:**
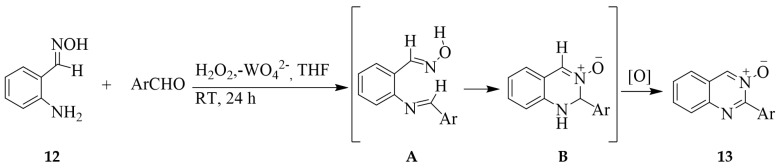
One-pot synthesis of quinazoline 3-oxides from 2-aminobenzaldoxime and benzaldehyde derivatives.

The 2-aminobenzaldoxime **12** was subjected to a one-pot reaction with benzaldehyde derivatives in the presence of H_2_O_2_-sodium tungstate in THF to afford after 24 h the corresponding quinazoline 3-oxides **13** in good overall yields (69–81%) [19]. The mechanism of this reaction is envisaged to involve the initial nucleophilic addition of the aniline derivative to the carbaldehyde group followed by cyclocondensation of the intermediate Schiff base **A** to afford a dihydroquinazoline 3-oxide derivative **B** (Scheme 5). H_2_O_2_-sodium tungstate then serves as an oxidizing system on **B** to afford **13**.

**Scheme 6:**
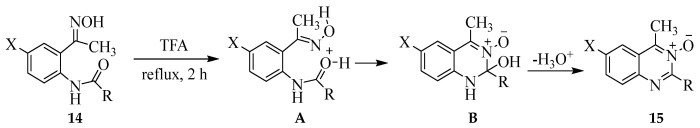
TFA-mediated intramolecular cyclization of oximes **14**.

Series of *N*-[2-(1-hydroxyiminoethyl)phenyl]amides and *N*-(4-halo-2-(1-(hydroxyimino)ethyl)phenyl)amide derivatives **14** (R = alkyl or aryl) were subjected to acid promoted intramolecular cyclization with trifluoroacetic acid (TFA) under reflux for 2 h to afford upon aqueous workup and purification through silica gel column chromatography the corresponding 2,4-dicarbo substituted quinazoline 3-oxides **15** in 72–89% yield (Scheme 6) [20]. These compounds resulted from initial protonation of the amide oxygen by TFA followed by an attack of the activated amide carbon of **A** to form intermediate **B**. The heterocyclic ring of the latter underwent spontaneous dehydrogenation to afford a fully aromatic derivative. These compounds were, in turn, evaluated through enzymatic assays (in vitro and in silico) for potential inhibitory effect against cyclooxygenase-1/2 (COX-1/2) and lipoxygenase-5 (LOX-5) activities as well as for free radical scavenging potential and cytotoxicity. Structure–activity relationship analysis suggested that the presence of a halogen atom at the C-6 position and a 2-aryl group enhanced the inhibitory effect against COX-2, and this observation was well supported by molecular docking studies. The presence of a π-electron delocalizing group on the fused benzo ring, on the other hand, enhanced the free radical scavenging effect of the quinazoline 3-oxides.

**Scheme 7:**
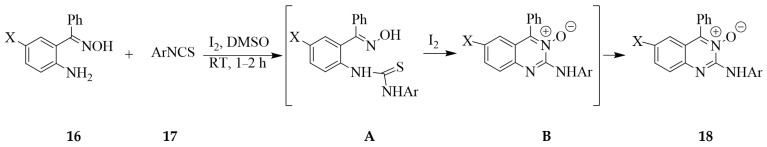
Iodine-mediated synthesis of the *N*,4-disubstituted quinazoline 3-oxide.

Methods that employ aryloximes and isothiocyanate for the construction of quinazoline 3-oxides derivatives in the presence of iodine have also been developed. The (2-aminophenyl)(phenyl)methanone oximes **16** (X = H or Cl) and arylisothiocyanates **17**, for example, were reacted with iodine in dimethylsulphoxide (DMSO) at RT to afford the corresponding 2-(arylamino)-4-phenylquinazoline 3-oxide derivatives **18** in 94–98% yield (Scheme 7) [21]. This reaction proceeded via the initial condensation of the oxime **16** with arylisothiocyanate **17** in DMSO to afford the thiourea intermediate **A**. Iodine-mediated cyclization of **A** afforded intermediate **B** via N–C and S–I bond formation. Aromatization of the latter intermediate occurred with the generation of HI and S to afford the cyclized aromatic products **18**. The analogous oximes derived from 2-aminoacetophenone [22] or 2-amino-5-bromo-3-iodoacetophenones [23], on the other hand, have previously been found to undergo methanesulfonyl chloride-mediated cyclization in the presence of triethylamine in dichloromethane at RT to afford the corresponding 1*H*-indazoles. Under similar reaction conditions, the *N*-aryl *o*-aminoacetophenone oximes afforded a variety of *N*-aryl-1*H*-indazoles and the analogous benzimidazoles when 2-aminopyridine and trimethylamine were used as bases, respectively [24].

**Scheme 8:**
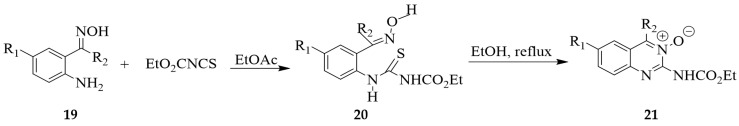
Synthesis of quinazoline oxide carbamates.

The oxime derivatives **19** were reacted with ethoxycarbonyl isothiocyanate in ethyl acetate to form the intermediate thioureas **20**, which spontaneously yclized in refluxing ethanol to afford the desired substituted ethyl (3-oxido-2-quinazolinyl)carbamates **21** in good yields (Scheme 8) [25].

**Scheme 9:**
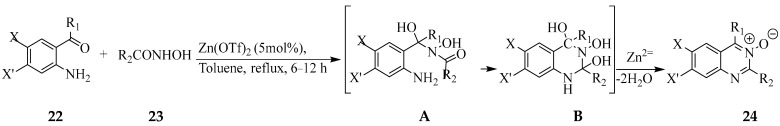
One-step synthesis of 2,4-disubstituted quinazoline 3-oxides **24**.

Madabhushi et al. previously employed Zinc(II) triflate (Zn(OTf)_2_) as a Lewis acid catalyst in anhydrous toluene under reflux to affect the cyclocondensation of 2-aminoaryl ketones **22** with acetohydroxamic acid derivatives **23** to afford the corresponding 2,4-disubstituted quinazoline 3-oxides **24** (Scheme 9) [26]. The mechanism of this reaction involves an initial attack of the electrophilic carbonyl carbon of 2-aminoacetophenone by the acetohydroxamic acid derivative to generate intermediate **A**. The latter would then undergo rapid intramolecular cyclization through the reaction of *N*-acetyl carbonyl with adjacent amine moiety followed by dehydration of **B** with the assistance of zinc species as a Lewis acid to produce a quinazoline 3-oxide with the elimination of two molecules of water [26].

Methods involving the use of transition metals for the synthesis of quinazoline 3-oxides have also been developed, and examples are discussed in the next section.

#### 2.2.2. Transition Metal-Mediated Reactions to Afford Quinazoline *N*-Oxides

**Scheme 10:**
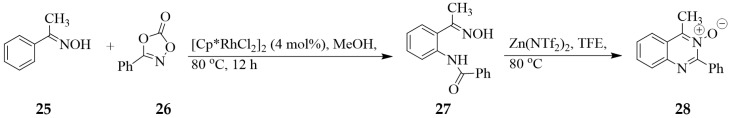
Amidation of keto oxime with 1,4,2-dioxazol-5-ones and Zn(II) catalyzed cyclization.

The *N*-(2-(1-(hydroxyimino)ethyl)phenyl)benzamide **27** was prepared as a sole product by subjecting acetophenone oxime **25** and 1,4,2-dioxazol-5-one **26** to dichloro(pentamethylcyclopentadienyl)rhodium(II) dimer ([Cp*RhCl_2_]_2_) as a catalyst in methanol under reflux for 12 h (Scheme 10) [27]. Attempted cyclization of this *N*-(2-(1-(hydroxyimino)ethyl)phenyl)benzamide in acetic acid by these authors resulted in the recovery of the starting material with no quinazoline 3-oxide detected in the reaction mixture. The keto oxime **25** was found to undergo Zn(II)-catalyzed cyclocondensation-dehydration in tetrafluoroethylene (TFE) under a nitrogen atmosphere at 80 °C in a pressure tube to afford the quinazoline 3-oxide **28** in yield of 93% [27].

**Scheme 11:**
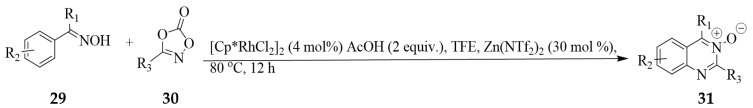
One-pot synthesis of quinazoline 3-oxides from ketoximes and 1,4,2-dioxazol-5-ones.

A one-pot Rh(III)-catalyzed C−H activation-amidation of the ketoximes **29** and 1,4,2-dioxazol-5-ones **30**, and subsequent Zn(II) catalyzed cyclocondensation-dehydration of the incipient *N*-(2-(1-(hydroxyimino)ethyl)phenyl)benzamide afforded the 2,4-dicarbo substituted quinazoline 3-oxides **31** (Scheme 11) [27]. The active RhCp*X_2_ (X = NTf_2_ or OAc) species is envisaged to be generated from the anion exchange between [RhCp*Cl_2_]_2_ and Zn(NTf)_2_ or HOAc.

**Scheme 12:**
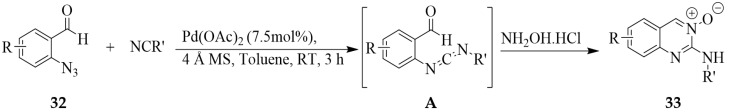
Palladium-catalyzed synthesis of substituted quinazoline 3-oxides **33**.

Sawant et al. developed a direct one-pot, three-component reaction of 2-azidobenzaldehyde, isocyanide, and hydroxylamine hydrochloride to afford quinazoline-3-oxides [10]. Palladium acetate catalyzed reaction of 2-azidobenzaldehyde **32**, isocyanides, and hydroxylamine hydrochloride in toluene in the presence of 4 Å molecular sieves under reflux afforded the quinazoline-3-oxides **33** in a single-pot operation (Scheme 12). The mechanistic study revealed that the reaction proceeds via initial palladium-catalyzed azide–isocyanide denitrogenative coupling to afford intermediate **A**. Oximation of the carbaldehyde moiety of this intermediate and subsequent 6-exo-dig cyclization afforded the quinazoline 3-oxide derivative. The addition of 4 Å molecular sieves improved the overall yield of the desired product by removing water produced in situ during the formation of hydrazine. Although several substituted isocyanides reacted well under these conditions, the aromatic and secondary isocyanides failed to react, and the starting materials were recovered unchanged.

Another conventional approach for the synthesis of quinazoline 3-oxides involves the dehydrogenation of the corresponding readily accessible 1,2-dihydroquinazoline 3-oxides [26], as described below.

### 2.3. Dehydrogenation of the 1,2-Dihydroquinazoline 3-Oxides

**Scheme 13:**
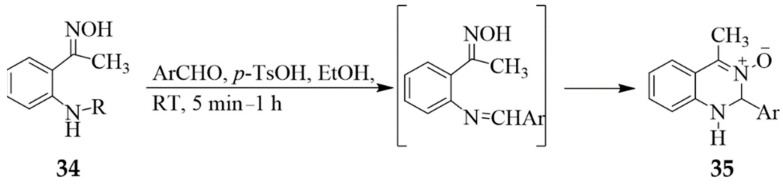
Synthesis of the 1,2-dihydroquinazoline 3-oxides **35**.

2-Aminobenzaldehyde or 2-aminoacetophenone derivatives readily undergo oximation with hydroxylamine hydrochloride in the presence of an amine base to afford the corresponding 2-aminobenzaldoximes or 2-aminoacetophenone oxime derivatives, respectively. Nucleophilic addition of the *o*-aminobenzaldoximes [28] or 2-aminoacetophenone oxime derivatives [29,30] to benzaldehyde derivatives and subsequent in situ cyclocondensation of the resultant intermediate afforded the corresponding 1,2-dihydroquinazoline 3-oxides. The latter were, in turn, evaluated for cytotoxicity against the human promyelocytic leukaemia HL-60 and lymphoblastic leukaemia NALM-6 cell lines [30]. The oxime derived from 2-aminoacetophenone **34** (R = H), for example, has previously been reacted with a series of aryl aldehydes in the presence of *p*-toluene sulfonic acid as a catalyst in ethanol at RT for 5–15 min. to afford the corresponding 1,2-dihydroquinazoline 3-oxides **35** (Scheme 13) [29]. Under similar reaction conditions, the oxime derived from 2-(methylamino)acetophenone (R = CH_3_) afforded after 1 h, the corresponding 1,2-dihydroquinazoline 3-oxides [31]. Samandran et al. also synthesised a series of the 1,2-dihydroquinazoline 3-oxides from the reaction of equimolar amounts of amino oximes with the corresponding aldehydes in ethanol at RT for 24 h [19].

**Scheme 14:**
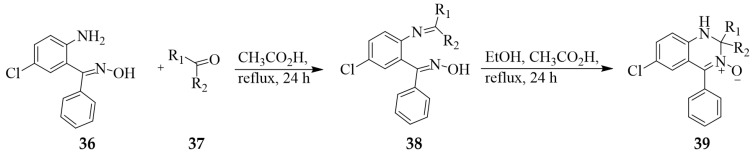
Synthesis of quinazoline 3-oxides **39**.

2-Aminoacetophenone oxime analogue **36** was previously reacted with butanedione monooxime **37** in acetic acid under reflux for 24 h to afford ketoximes **38** (Scheme 14) [30]. The latter were, in turn, cyclized in ethanol–acetic acid mixture under reflux for 24 h to afford the corresponding quinazoline 3-oxides **39** in 60–75% yield (Table 1). These quinazoline 3-oxides were evaluated for cytotoxic activities against the human leukaemia HL-60 cells under hypoxic and aerobic conditions using tirapazamine as the reference standard.

**Scheme 15:**
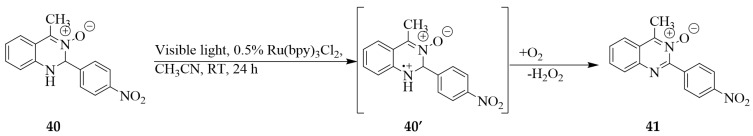
Light-induced dehydrogenation of a 1,2-dihydroquinazolin 3-oxide derivative.

Chen and Yang previously exposed 4-methyl-2-(4-nitrophenyl)-1,2-dihydroquinazoline-3-oxide **40** to visible light in the presence of 0.5 mol % tris(bipyridine)ruthenium(II) chloride (Ru(bpy)_3_Cl_2_) as a photocatalyst in acetonitrile under aerobic conditions and isolated 4-methyl-2-(4-nitrophenyl)quinazoline 3-oxide **41** in 63% yield (Scheme 15) [29]. No product was obtained when the photooxidation of **40** was conducted under argon atmosphere prompting the authors to suggest the importance of molecular oxygen as the oxidant for this photoreaction. It is envisaged that visible light excited the Ru(bpy)_3_^2+^ to accept one electron from NH of **40** to yield the cation radical **40′** and the Ru(bpy)_3_^+^ (see ref [29] for fragmentation pattern). Electron transfer from the latter to molecular oxygen yielded the superoxide anion radical and regenerated the ground-state photocatalyst Ru(bpy)_3_^2+^. It is envisaged that the cation radical **40′** underwent proton and hydrogen transfers to the superoxide anion radical to furnish the quinazoline 3-oxide **41** extruding hydrogen peroxide as a by-product. 

**Scheme 16:**
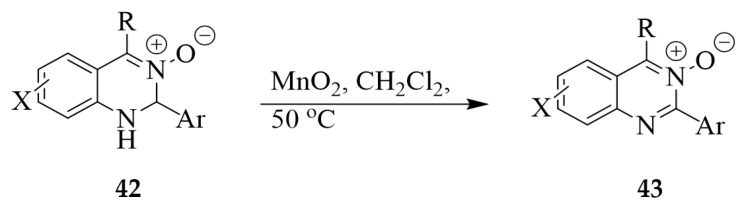
Active manganese oxide-mediated dehydrogenation of **43**.

Oxidizing agents such as 2,3-dichloro-5,6-dicyanobenzoquinone (DDQ) [32], active manganese oxide (MnO_2_) [8], and hydrogen peroxide (H_2_O_2_)-tungstate [19] were used before to transform the dihydroquinazoline 3-oxides into the corresponding quinazoline 3-oxides. A series of quinazoline 3-oxides **42** (X = H, 6-Cl/Br or 7-Me) substituted at the 2-position with an alkyl or benzyl group and an electron donating or withdrawing group at the 4-position (alkyl, aryl, or heteroaryl) were synthesized in good to excellent yields (54–88%) by oxidation of the corresponding 1,2-dihydroquinazoline 3-oxides **43** using 3 equiv. of activated MnO_2_ in dichloromethane at 50 °C (Scheme 16) [8]. The advantage of the use of MnO_2_ as an oxidant is the ease of its removal from the reaction mixture which involves simple filtration.

**Scheme 17:**
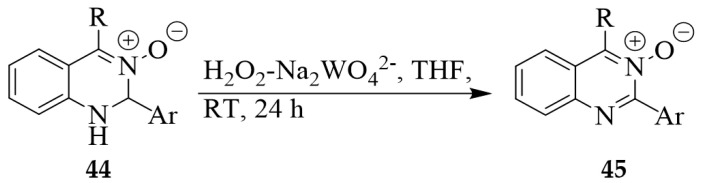
H_2_O_2_-sodium tungstate mediated dehydrogenation of dihydroquinazoline 3-oxides.

Coșkun et al. have also dehydrogenated the dihydroquinazoline 3-oxides **44** using H_2_O_2_-sodium tungstate oxidant system in THF to afford after 24 h at RT the corresponding quinazoline 3-oxides **45** (Scheme 17) [33]. However, the one-pot synthesis of these quinazoline 3-oxides from the 2-aminobenzaldoximes (refer to Scheme 5) proceeded in a relatively short time resulting in improved overall yields [19].

### 2.4. Chemical Transformation of Quinazoline 3-Oxides

Quinazolines *N*-oxides can undergo deoxygenation into quinazolines [34], acetoxylation [29] and ring expansion to benzodiazepines [8,25].

#### 2.4.1. Deoxygenation of Quinazoline *N*-Oxides

**Scheme 18:**
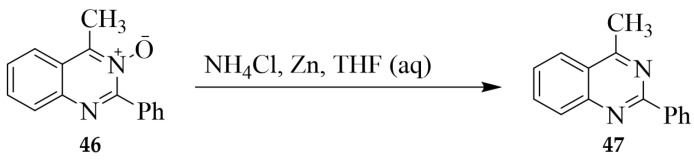
Zn-catalyzed deoxygenation of 4-methyl-2-phenylquinazoline 3-oxide **46**.

**Scheme 19:**
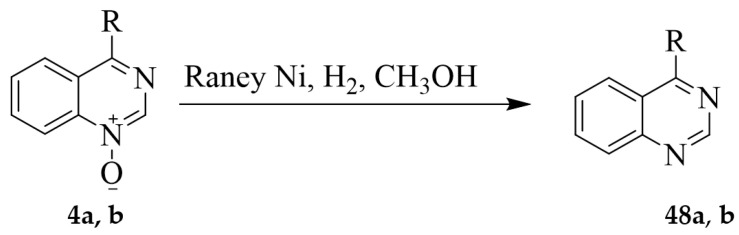
Raney nickel catalysed deoxygenation of **4a** and **4b**.

The N–O bond in pyrimidine *N*-oxides is cleaved by catalytic reduction, low-valent phosphorus (PCl_3_ or POCl_3_) or titanium (TiCl_3_) reagents, as well as by the more common metals used for hydrogenolysis. Deoxygenation of 4-methyl-2-phenylquinazoline 3-oxide **46** using Zn in the presence of aqueous NH_4_Cl in THF afforded 4-methyl-2-phenylquinazoline **47** in 71% yield (Scheme 18) [26]. Deoxygenation of the analogous quinazoline 1-oxides **4a** (R = CH_3_) and **4b** (R = -CH_2_CH_3_), on the other hand, was achieved through catalytic hydrogenation (Raney Ni catalyst in MeOH under hydrogen (H_2_) stream) to afford 4-substituted quinazoline **48** in 33–43% yield (Scheme 19) [11].

**Scheme 20:**
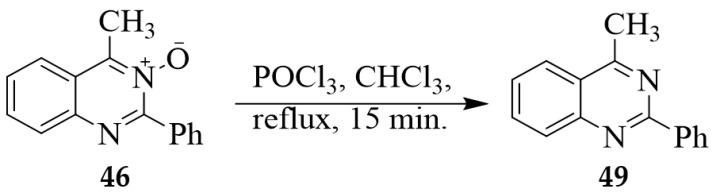
POCl_3_-mediated reduction of quinazoline 3-oxide **46**.

A mixture of *N*-oxide **46** and phosphorus oxychloride in chloroform was heated at reflux for 15 min. followed by aqueous work-up and purification through silica gel column chromatography to afford **49** in 18% yield (Scheme 20) [15]. Improved yield (70%) of this quinazoline derivative was observed when this quinazoline 3-oxide was treated with PCl_5_ in dichloromethane at RT for 15 min. [30].

#### 2.4.2. Alkoxylation of Quinazoline *N*-Oxides

**Scheme 21:**
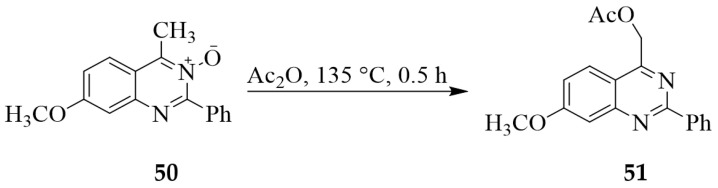
Alkoxylation of **50** with acetic anhydride under reflux.

The highly acidic proton of the methyl group at the C-4 position of quinazoline-3-oxide scaffold has been found to promote acetoxylation to ester derivatives. 4-Methyl-7-methoxy-2-phenyl substituted quinazoline 3-oxide **50**, for example, was subjected to acetic anhydride under reflux for 0.5 h to afford the ester derivative **51** in 82% yield (Scheme 21) [27].

#### 2.4.3. Alkylation of Quinazoline *N*-Oxides

**Scheme 22:**
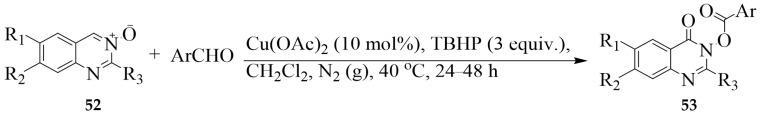
Copper-catalyzed oxidative coupling of quinazoline 3-oxide and benzaldehydes.

*N*-Oxide moiety in aza-heteroarene represents an efficient and removable directing group for *ortho* C–H bond activation. Zhao et al., for example, effected a copper-catalyzed oxidative coupling reaction between Csp^2^–H of quinazoline 3-oxide **52** and Csp^2^–H of benzaldehyde derivatives in the presence of *tert*-butyl hydroperoxide (TBHP) in dichloromethane at 40 °C under nitrogen atmosphere to furnish the quinazolinone derivatives **53** (Scheme 22) [34]. Both aliphatic and aromatic substituents at the 2-position of the quinazoline 3-oxide scaffold were tolerated though the yields decreased with the increase of the chain length from the methyl to the propyl group. α,β-Unsaturated aldehydes, heteroaryl aldehydes, and aliphatic aldehydes were also found to be suitable acyl donors to afford cyclic hydroxamic esters in good to excellent yields.

**Scheme 23:**
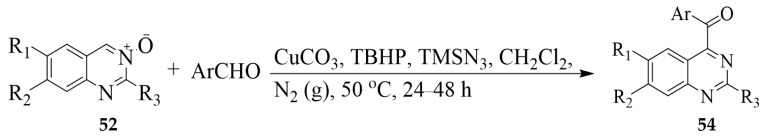
Formation of quinazoline aryl ketones in the presence of CuCO_3_–TMSN_3_ mixture.

The authors observed the formation of quinazoline aryl ketone derivatives **54** from **52** in the presence of Cu(OAc)_2,_ albeit in low yields when the reaction was quenched prematurely. The quinazoline aryl ketones **54** were isolated as sole products in the presence of trimethylsilyl azide (TMSN_3_) and copper carbonate (CuCO_3_) (Scheme 23). Controlled reactions revealed that compounds **53** are the consequence of initial in situ Baeyer–Villiger oxidation of quinazoline aryl ketones **54** followed by intramolecular acyl transfer to afford **53** [34].

**Scheme 24:**
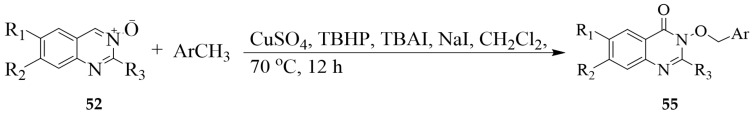
Oxidative coupling of quinazoline 3-oxides **52** and benzylic derivatives.

In a subsequent study, these authors employed this strategy on benzylic Csp^3^–H bonds with quinazoline 3-oxides **52** in the presence of CuSO_4_ (3 mol %), TBHP (2 equiv.), 20 mol % of tetrabutylammonium iodide (TBAI), and NaI (70 mol %) in dichloromethane at 70 °C in sealed tubes to afford after 12 h the corresponding quinazolinone derivatives **55** (Scheme 24) [35].

**Scheme 25:**
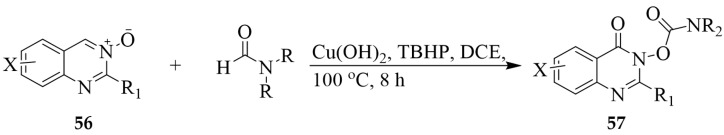
Copper-catalyzed oxidative coupling of quinazoline 3-oxides and formamides.

A copper-catalyzed oxidative coupling reaction between Csp^2^–H of quinazoline 3-oxide **56** and Csp^2^–H of formamides in the presence of copper hydroxide and TBHP in dichloroethane (DCE) also afforded the analogous *O*-quinazolinone carbamates **57** (Scheme 25) [36]. The latter are envisaged to be formed through a reaction sequence involving radical addition, Baeyer–Villiger oxidation, and intramolecular acyl transfer [36].

**Scheme 26:**
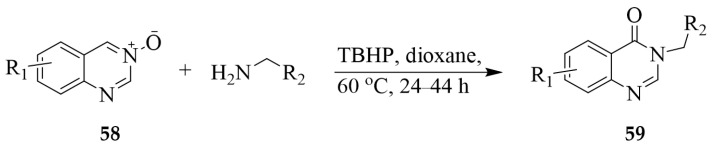
TBHP oxidative coupling of quinazoline 3-oxides and primary amines.

**Scheme 27:**
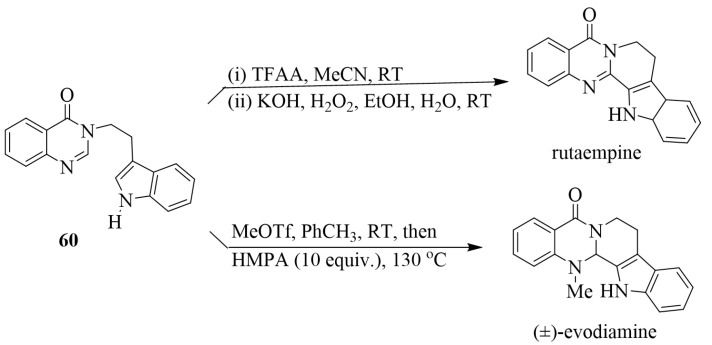
Synthesis of rutaempine and evodiamine from **60**.

Quinazoline 3-oxides **58** reacted with primary amines in the presence of TBHP as the oxidant in dioxane under reflux for 24–44 h to afford the quinazolin-4(3*H*)-one derivatives **59** (Scheme 26) [37]. The mechanism of this reaction was investigated using control reactions, and ESI-MS analysis revealed a complex reaction involving multiple bond dissociation/recombination steps. These mild reactions and metal-free conditions were found to be compatible with a broad range of primary amines, producing a series of quinoxaline-4(3H)-ones. Moreover, this methodology also afforded 3-(2-(1*H*-indol-3-yl) ethyl)quinazolin-4(3*H*)-one **60** in 70% yield, which is a precursor for the synthesis of bioactive rutaempine and (±)-evodiamine (Scheme 27) [37].

**Scheme 28:**
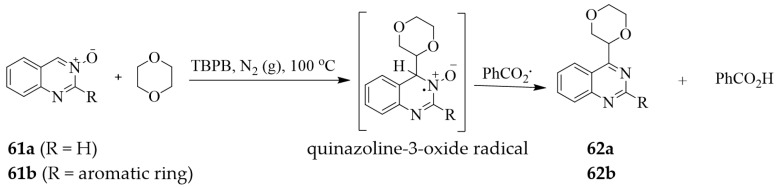
Cross-coupling of quinazoline-3-oxides **61a** and **61b** with various ethers.

Direct C-4 alkylation of the 2-unsubstituted and 2-aryl substituted quinazoline-3-oxides was previously achieved with open chain (1,2-dimethoxyethane, diethoxymethane or diethyl ether) or cyclic ethers (1,3-dioxolane or 1,3-benzodioxole) in the presence of *tert*-butyl peroxybenzoate (TBPB) afforded series of oxidative cross-coupling products in moderate to good yields [38]. Scheme 28 shows the reactions of 1,4-dioxane with **61a** (R = H) and **61b** (R = Ar) as representative models for the radical oxidative cross-coupling reaction of the sp^3^ C–H bond in ethers with the sp^2^ C–H bond in quinazoline-3-oxide to afford **62a** and **62b**, respectively. The mechanism of this radical oxidative cross-coupling reaction is envisaged to involve the initial decomposition of TBPB to generate a *tert*-butoxyl radical and a benzoate radical. The most reactive *tert*-butoxyl radical then abstracted hydrogen from 1,4-dioxane, and the resultant dioxane radical added to quinazoline 3-oxide **61** to generate a quinazoline-3-oxide radical. Abstraction of a hydrogen atom from the quinazoline-3-oxide radical by less sterically hindered benzoate radical (versus *tert*-butoxyl radical) afforded quinazoline-3-oxide **62** and benzoic acid as a by-product [38].

**Scheme 29:**
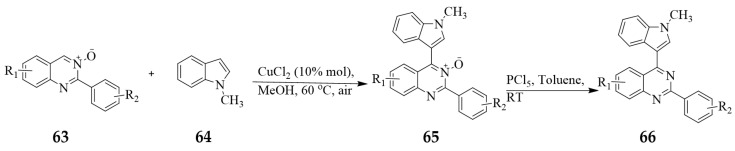
Copper-catalyzed cross-dehydrogenative coupling of quinazoline 3-oxides and indoles.

Copper(II) chloride has been employed to catalyze the Csp^2^–H bond (C-4) of the 2-aryl substituted quinazoline-3-oxide **63** and the Csp^2^–H bond (C-3) of various indoles **64** to facilitate the cross-dehydrogenative-coupling reaction between these two chromophores to afford the quinazoline 3-oxide appended indole hybrids **65** (Scheme 29) [39]. The *N*-methylindoles substituted with alkyl, halide, or alkoxy group at the 5-position afforded the expected quinazoline 3-oxide–indole hybrids in moderate to good yields. However, indoles substituted on nitrogen with an electron-withdrawing group, such as the tosyl group, failed to react. Subsequent dehydrogenation of these molecular hybrids with PCl_5_ (1.2 equiv.) in toluene at RT afforded quinazoline-indole hybrids **66**.

Quinazoline 3-oxides are valuable intermediates in the synthesis of benzodiazepine analogues [8] and other polycyclic compounds of biological importance [9], and examples of these reactions are described in the next sections. The analogous 4-(1-benzyl-1*H*-indol-3-yl)-6,7-dimethoxyquinazoline has previously been found to exhibit moderate activity against protein tyrosine kinase ErbB-2, with little or no activity against the epidermal growth factor receptor tyrosine kinase (EGFR-TK) [40]. The 4-(indole-3-yl)quinazolines, on the other hand, were found to be highly potent EGFR-TK inhibitors with excellent cytotoxic properties against several cancer cell lines [41].

### 2.5. Synthesis of Polycyclic Quinazoline Derivatives and Benzodiazepine Analogues

**Scheme 30:**
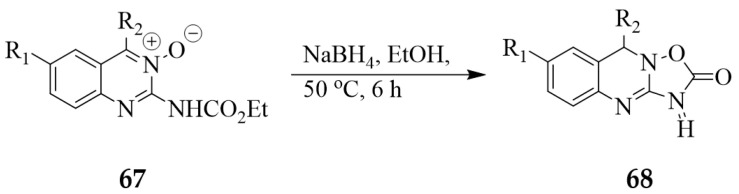
Synthesis of polycyclic quinazolines.

The oxygen atom of quinazoline 3-oxides can also participate in ring-closure reactions to yield polycyclic derivatives. The 3-oxidoquinazoline-2-carbamates 67, for example, were found to undergo reductive ring closure to afford the 3,9-dihydro-2*H*-[1,2,4]oxadiazolo[3,2-*b*]quinazolin-2-ones **68** (Scheme 30) [26].

**Scheme 31:**
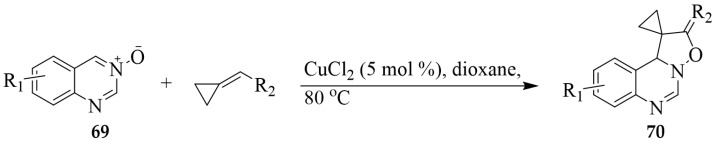
[3 + 2] cycloaddition of quinazoline 3-oxide with alkylidenecyclopropane derivatives.

Wu *et al.* reported a copper-catalyzed [3 + 2] cycloaddition of quinazoline 3-oxides **69** with alkylidenecyclopropane derivatives to afford the angular polycyclic quinazoline derivatives **70** (Scheme 31) [42]. Methyl, methoxy, fluoro and chloro functionalities were all tolerated, leading to the formation of the corresponding *N*-(2-(5-oxa-6-azaspiro[2.4]hept-6-en7-yl)phenyl) **69** in high yield.

**Scheme 32:**
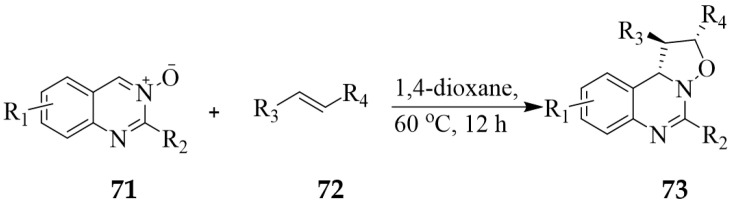
[3 + 2] Cycloaddition between quinazoline 3-oxide and methyl 3-methoxyacrylate.

Yin et al. studied the [3 + 2] cycloaddition reaction between the quinazoline 3-oxides **71** (R_2_ = H, alkyl, aryl) and various alkene derivatives **72** such as methyl 3-methoxyacrylate (R_3_ = -CO_2_Me, R_4_ = -OMe), ethyl-3-ethoxyacrylate (R_3_ = -CO_2_Et, R_4_ = -OEt), dimethyl maleate (R_3_, R_4_ = -CO_2_Me), acrylonitrile (R_3_ = -CN, R_4_ = H) and 5-methyl-hex-2-enoic acid methyl ester (R_3_ = -CO_2_Me, R_4_ = -CH_2_CH(CH_3_)_2_) to afford a series of isoxazolo[2,3*-c*]quinazoline **73** in good to excellent yield with total regio- and stereoselectivities (Scheme 32) [9]. A density functional theory (DFT) method using the B3LYP/6-31G(d) basis set further predicted the reaction to be under thermodynamic control and to favour exclusive formation of the *ortho-exo* cycloadduct in agreement with experimental finding [43]. Hitherto, Heaney et al. reacted 2-styrylquinazoline 3-oxide **71** (R_2_ = -CH=CHPh) with phenyl vinyl sulfone or *N*-methyl maleimide in THF under reflux and isolated the corresponding isoxazolo[2,3-*c*]quinazoline derivatives in very low yields [44]. These tricyclic compounds were found to be unstable in solution at RT and to rearrange to other complex products.

**Scheme 33:**
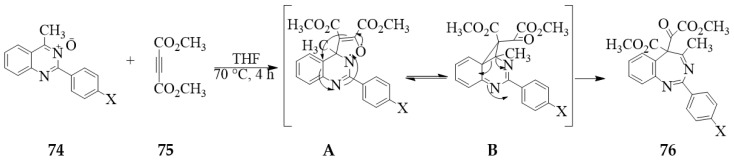
DMAD assisted ring expansion of **74** into benzodiazepine analogues **76**.

The use of acetylene derivatives as dipolarophiles on the 4-carbo substituted quinazoline 3-oxides, on the other hand, resulted in the isolation of benzodiazepine analogues instead of the polycyclic quinazoline derivatives. The 2-aryl-4-methylquinazoline 3-oxides **74**, for example, were reacted with dimethyl acetylenedicarboxylate (DMAD) **75** in THF at 70° for 4 to afford the methyl 5-(2-methoxy-2-oxoacetyl)-4-methyl-2-phenylsubstituted-5*H*-benzo[*d*][1,3]diazepine-5-carboxylates **76** in 67–74% yield (Scheme 33) [8]. The formation of these benzodiazepine derivatives is envisaged to proceed via the rearrangement of the incipient tricyclic quinazoline intermediate **A** as represented in the Scheme.

**Scheme 34:**
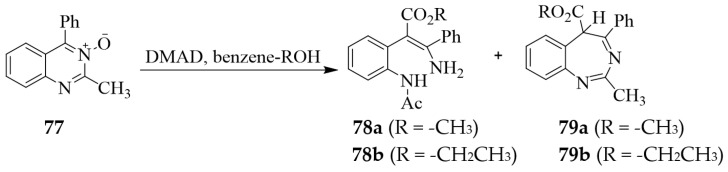
Reaction of 2-methyl-4-phenylquinazoline 3-oxide **77** with DMAD.

DMAD, on the other hand, reacted with the isomeric 2-methyl-4-phenylquinazoline 3-oxide **77** in benzene-(m)ethanol followed by purification on basic alumina to afford the phenyl acrylates **78** (13–21%) and the potentially tautomeric benzodiazepines **79** in 5–14% yield together with smaller amounts of other products (Scheme 34) [28].

**Scheme 35:**
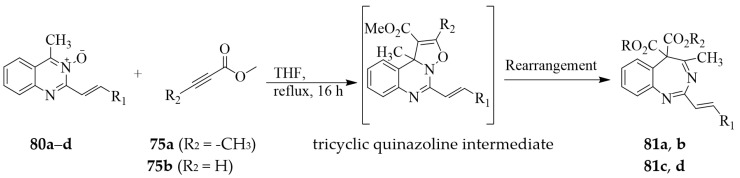
Cyclization of **80a**–**d** with DMAD **75a** or methyl propiolate **75b**.

The analogous 4-methyl-2-styrylquinazoline 3-oxides **80a**–**d** were also reacted with dimethyl acetylenedicarboxylate (DMAD) **75a** (R_2_ = -CO_2_CH_3_) or methyl propiolate **75b** (R_2_ = H) as dipolarophiles (2 equiv.) in dry THF under reflux for 16 h followed through purification by column chromatography on either silica gel (SiO_2_) or neutral alumina (Al_2_O) to afford the corresponding benzodiazepine analogues **81a**–**d** (Scheme 35) [44]. The presence of the 2-styryl group resulted in significantly reduced yields of the corresponding benzodiazepine derivatives compared to the products of the reaction of analogous 2-phenyl-4-methylquinazoline 3-oxides **74** with DMAD (refer to Scheme 33 above). The heterocyclic ring of these compounds was hydrolysed during purification by column chromatography on either or both SiO_2_ or Al_2_O (Table 2) and the extend of hydrolysis depended on the nature of the C-4 and C-5 substituents on the diazepine ring. The presence of the carbon-carbon double bond in the five-membered ring of the tricyclic quinazoline intermediate implicated in the reaction of the 4-carbo substituted quinazoline 3-oxides **74**, **77** or **80** with acetylene derivative **75a** or **75b** facilitated the rearrangement and subsequent ring enlargement to afford benzodiazepine analogues.

### 2.6. Ring Expansion of Quinazoline 3-Oxides to Afford Benzodiazepine Analogues

**Scheme 36:**
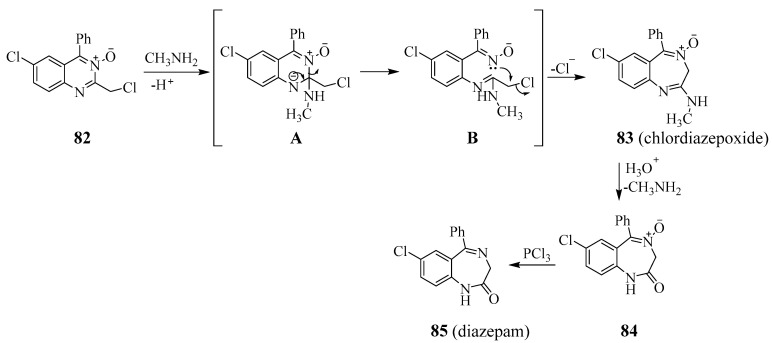
Reaction of methylamine with 2-chloromethyl quinazoline 3-oxide.

Nucleophilic attack on C-2 of 2-chloromethyl quinazoline 3-oxide **82** by methylamine followed sequentially by ring opening of intermediate **A** and intramolecular displacement of chlorine atom by nitrogen atom of the oxime moiety afforded chlordiazepoxide **83** as shown in Scheme 36 [26]. Acid hydrolysis of chlordiazepoxide and in situ hydrolysis of the benzodiazepin-2-one 4-oxide intermediate **84** followed by its PCl_3_ mediated deoxygenation afforded 1,4-benzodiazepine (diazepam) **85**. Benzodiazepines enhance the effect of the neurotransmitter gamma-amino butyric acid (GABA-A), resulting in sedative, hypnotic, anxiolytic, anticonvulsant and muscle relaxant properties [45]. These properties make benzodiazepines and their analogues useful drugs in the treatment of anxiety, insomnia, agitation, seizures, muscle spasms, alcohol withdrawal and as a premedication for medical or dental procedures. Chlordiazepoxide and diazepam, for example, are the central nervous system (CNS) agents used for the treatment of muscle spasms, seizures, trauma, and anxiety disorders [8].

## 3. Conclusions

Aromatic *N*-oxides are desirable biologically active compounds with a potential for application in pharmaceutical and agrochemical industries. It is imperative for medicinal chemists to continue to develop environmentally friendly and mild methods for the production of quinazoline 3-oxides. This scaffold is capable of undergoing various chemical transformations into biologically-relevant polysubstituted quinazolines and their polynuclear derivatives, as well as ring expansion to afford the benzodiazepine analogues with CNS activity. The potential for the quinazoline 3-oxide scaffold to undergo transition metal catalyzed cross-dehydrogenative-coupling, on the other hand, makes them suitable candidates for the design and synthesis of other novel biologically-relevant molecular hybrids. Moreover, the presence of a halogen atom on the fused benzo ring of the quinazoline 3-oxide framework would facilitate further chemical transformation via transition metal catalyzed cross-coupling reactions to afford polysubstituted derivatives. It is envisaged that this review will help medicinal chemistry researchers to design and synthesize new quinazoline 3-oxides and their derivatives and investigate their biological properties to treat various diseases.

## Figures and Tables

**Table 1:** Substitution pattern and percentage yields of quinazoline 3-oxides **39a**–**c**.

39a–c	R_1_	R_2_	% Yield
**39a**	H	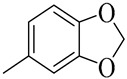	70
**39b**	H	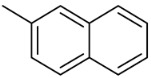	75
**39c**	CH_3_	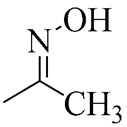	60

**Table 2:** Substitution pattern and percentage yields of benzodiazepines **81a**–**d**.

81a–d	R_1_	R_2_	% Yield (Purification)
**81a**	Ph	-CO_2_Me	36 (SiO_2_); 30 (Al_2_O)
**81b**	Me	-CO_2_Me	34 (SiO_2_); 32 (Al_2_O)
**81c**	Ph	H	23 (SiO_2_); 60 (Al_2_O)
**81d**	Me	H	7 (SiO_2_); 54 (Al_2_O)

## Data Availability

Not applicable.
